# Editorial Comment: Safety of a Novel Thulium Fiber Laser for Lithotripsy: An In Vitro Study on the Thermal Effect and Its Impact Factor

**DOI:** 10.1590/S1677-5538.IBJU.2020.05.07

**Published:** 2020-07-31

**Authors:** Alexandre Danilovic

**Affiliations:** 1 Hospital das Clínicas da Faculdade de Medicina da USP - HCFMUSP Serviço de Urologia São Paulo SP Brasil Serviço de Urologia, Hospital das Clínicas da Faculdade de Medicina da USP - HCFMUSP, São Paulo, SP, Brasil

Peng Y ^1^, Liu M ^1^, Ming S ^1^, Yu W ^2^, Li L ^1^, Lu C ^1^, et al.

## COMMENT

Thulium fiber laser (TFL) is composed of silica fiber doped with Thulium ions triggered during the laser activation. Since 2005, TFL has been evaluated in several preclinical studies for the management of urolithiasis (1). Previous in vitro studies showed TFL produced faster stone ablation rates, smaller stone fragments and lower retropulsion than the Holmium:YAG laser (2, 3). However, the specific heat production of TFL is four times higher than that of the Holmium:YAG laser (2). Irrigation rate and laser power settings are critical to avoid biological damage due to thermal effect of Holmium:YAG laser (4). Therefore, it is important to know the major determinants of thermal effect of TFL.

This in vitro study evaluated the impact of different TFL power settings and irrigation rates on water temperature. The investigators used a novel TFL prototype (Raykeen Laser Technology Limited Corporation, Shanghai, China) with a maximum power output of 55W in super pulse mode with 272 pm core diameter fiber. The safety threshold of temperature for laser surgery was established in 43° C. This study found TFL power > 15W may cause heat injury of tissues when irrigation is ceased during lithotripsy. TFL power up to 30 W was safe with a moderate irrigation rate of 15 mL/min. The authors recommend irrigation rate ≥ 25 mL/min or intermittent laser firing when using TFL power above 30W.

Although this study established in vitro safety irrigation rates and power settings for the thermal effect of TFL, the authors did not tested different fiber sizes. Fiber size influences irrigation rates. Therefore, future studies are needed to elucidate the thermal effect of TFL using smaller fibers and its influence using animal models.
